# Supplementary material for: Functional Roles of CD26/DPP4 in Bleomycin-Induced Pulmonary Hypertension Associated with Interstitial Lung Disease
Source: Int J Mol Sci. 2024 Jan 6;25(2):748. doi: 10.3390/ijms25020748 (PMC10815066; doi:10.3390/ijms25020748)
Supplement: Supplementary file 1 [file ijms-25-00748-s001.zip › ijms-2764087-supplementary.pdf]

(a)

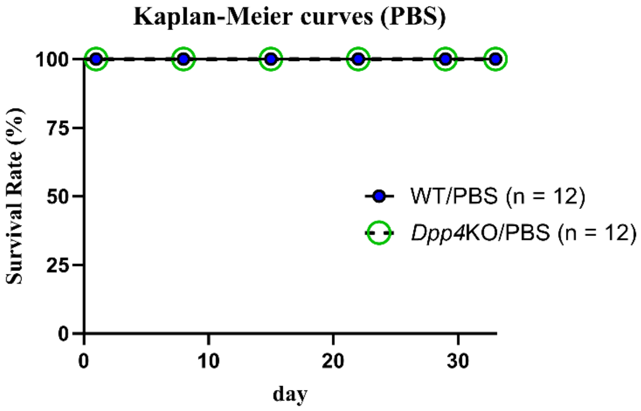

(b)

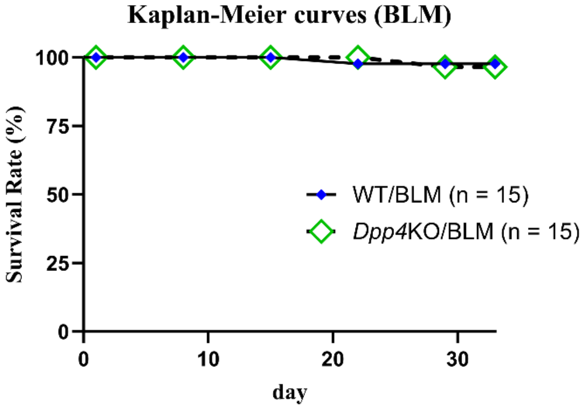

Supplemental Figure S1. Survival rates of WT and *Dpp4*KO mice until day 33 determined using the Kaplan–Meier method: (a) treatment with PBS, (b) treatment with BLM.

Supplemental Table S1. Details of RT-qPCR primers used in this study.

Gene	Forward Primer	Reverse Primer	Melting Temperature (°C) (Forward/ Reverse)
Dpp4	5′-CGGTATCATTTA GTAAAGAGGCAAA	5′-GTAGAGTGTAGA GGGGCAGACC	64.6 / 63.3
DPP4	5′-GCACGGCAACAC ATTGAA	5′-TGAGGTTCTGAA GGCCTAAATC	63.0 / 62.9
